# The response of early neural genes to FGF signaling or inhibition of BMP indicate the absence of a conserved neural induction module

**DOI:** 10.1186/1471-213X-11-74

**Published:** 2011-12-15

**Authors:** Crystal D Rogers, George S Ferzli, Elena S Casey

**Affiliations:** 1 Department of Biology, Georgetown University, Washington DC, USA

## Abstract

**Background:**

The molecular mechanism that initiates the formation of the vertebrate central nervous system has long been debated. Studies in *Xenopus *and mouse demonstrate that inhibition of BMP signaling is sufficient to induce neural tissue in explants or ES cells respectively, whereas studies in chick argue that instructive FGF signaling is also required for the expression of neural genes. Although additional signals may be involved in neural induction and patterning, here we focus on the roles of BMP inhibition and FGF8a.

**Results:**

To address the question of necessity and sufficiency of BMP inhibition and FGF signaling, we compared the temporal expression of the five earliest genes expressed in the neuroectoderm and determined their requirements for induction at the onset of neural plate formation in *Xenopus*. Our results demonstrate that the onset and peak of expression of the genes vary and that they have different regulatory requirements and are therefore unlikely to share a conserved neural induction regulatory module. Even though all require inhibition of BMP for expression, some also require FGF signaling; expression of the early-onset pan-neural genes *sox2 *and *foxd5α *requires FGF signaling while other early genes, *sox3*, *geminin *and *zicr1 *are induced by BMP inhibition alone.

**Conclusions:**

We demonstrate that BMP inhibition and FGF signaling induce neural genes independently of each other. Together our data indicate that although the spatiotemporal expression patterns of early neural genes are similar, the mechanisms involved in their expression are distinct and there are different signaling requirements for the expression of each gene.

## Background

Development of the vertebrate central nervous system (CNS) is initiated during gastrulation when dorsal ectodermal cells are converted to the neural fate. There are two prevailing models for the induction of the CNS. The first, the neural default model, arose from experiments demonstrating that in the absence of bone morphogenetic protein (BMP) signaling, amphibian ectodermal explants form neural tissue instead of epidermis [1,2]. Formation of the nervous system by default is highly conserved. In the protostome *Drosophila melanogaster*, neural tissue forms as a result of inhibition of the BMP homolog Decapentaplegic (Dpp) by the Chordin ortholog Sog [3]. Furthermore, the *Xenopus *BMP antagonist Noggin is sufficient to inhibit Dpp and induce neuroectoderm in fruit flies [4], and overexpression of Sog induces a secondary axis in *Xenopus *embryos [5]. The second model, the instructive signaling model, arose from studies of chick embryonic development and indicated that inhibition of BMP signaling is not sufficient to induce neural tissue, and that instruction from another signaling pathway such as FGF (fibroblast growth factor), is required. There is evidence supporting both models in multiple vertebrates [6,7], thus feeding the controversy over which signals are necessary and sufficient during vertebrate CNS induction.

Experiments in ES cells, mouse and zebrafish embryos support the model that the vertebrate CNS is formed by default. Like *Xenopus *ectodermal explants [8], mouse ES cells [9], and human ES [10-12] and induced pluripotent stem cells [11] are converted to rostral neural tissue when BMP signaling is inhibited. In the mouse epiblast, BMP2/4 signaling maintains pluripotency and prevents the acquisition of a neural fate, whereas inhibition of BMP signaling induces neural tissue independent of FGF signaling [13]. Similarly, in zebrafish, BMP inhibition is sufficient for the induction of anterior neural genes and FGF signaling is not required for induction but rather for posteriorization of the induced tissue [14].

Although BMP inhibition clearly plays a role in neural specification in many organisms, alone it does not effectively induce neural tissue formation in *Xenopus *ventral ectoderm [15-17] or outside of the chick dorsal ectoderm [18], which indicates that an instructive signal is required. The leading candidate for this instructive signal is FGF. In both chick and frog, overexpression of FGF2, FGF4, or FGF8 induces the expression of posterior neural genes [18-21] and the activation of FGF signaling in combination with BMP antagonism induces the expression of pan-neural genes in non-neural ectodermal territories [17,19]. Loss of function studies in mESCs, chick and *Xenopus *embryos also suggest a role for instructive signaling by FGF in neural induction. For example, inhibition of FGF receptors or Erk1/2 by exposure to pharmacological inhibitors eliminated differentiation of mESCs into neurons [22] and resulted in a loss of neural tissue in frog [15], zebrafish [23] and chick [24]. Furthermore, overexpression of the dominant negative FGF receptor 4a reduced the expression of the neural progenitor marker, *sox2*, in tailbud-stage *Xenopus *embryos [15] and the formation of neural tissue in ectodermal explants in response to Noggin [25].

It has been difficult to dissect out an independent role for FGF in neural induction because FGF signaling has significant roles in mesodermal development [26] and neural anterior-posterior patterning [27], induces neural tissue via interference with BMP transcription and transduction [26-28] and has been proposed to maintain rather than induce a neural progenitor population [29-32]. Specifically, studies suggest that neural specification in response to FGF is not instructive or independent from BMP inhibition but rather, is the result of interference with BMP signaling via inhibition of Smad1 activity [28] or the transcription of BMP [29,30]. It has also been proposed that FGF signaling is dispensable for induction and instead is required for the maintenance of neural progenitors. This is supported by studies in: (1) *Xenopus *ectodermal explants in which *sox2 *and *sox3 *expression is not maintained when FGF signaling through FGFR1 and 2 is inhibited [31]; (2) the mouse olfactory bulb and retina in which a proliferating progenitor population is decreased in the absence of FGF signaling [32,33] and; (3) hESCs in which exogenous FGF maintains cells in an undifferentiated state [34]. Furthermore, the approaches and techniques used to investigate the role of FGF have been called to question. It has been argued that incorrect markers were analyzed at the wrong developmental stages and in the wrong tissues, and that doses of pharmacological inhibitors were lethal or detrimental to development [8]. With this conflicting data and the variables added by the use of many different model organisms, stages, neural markers and FGF inhibitors (small molecules, dominant negative receptors, morpholinos), it remains unclear if FGF signaling is required in addition to, or independent of, BMP inhibition for the induction of neural genes.

This study compares the regulatory requirements for the onset and maintenance of multiple early neural genes in *Xenopus *embryos. Using multiple genes in one organism has allowed us to determine whether FGF signaling is required for the induction of multiple early neural genes independent of BMP inhibition, and also to determine the role of FGF8a in neural development. Using gain and loss of function assays, we show that *sox2 *and *foxd5a *require FGF signaling for neural induction and that *sox3 *and *geminin *require FGF signaling for maintenance of expression. Ultimately, we show that depending on the gene of interest, the ability of FGF to induce expression can be dependent on the absence of BMP signaling and may be indirect via the induction of mesoderm.

## Results

### Response of early neural genes to BMP inhibition and FGF signaling

**Figure 1:**
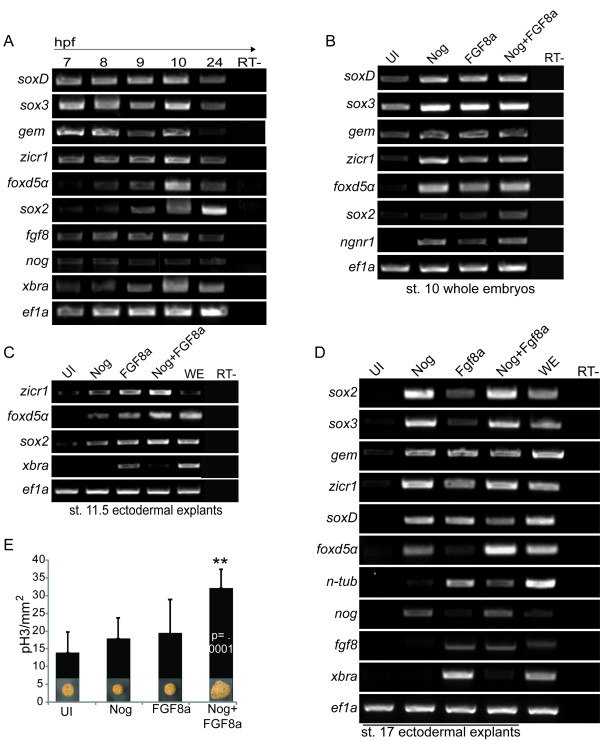
**Temporal expression of early neural genes and response to Noggin and FGF8a**. Semi-quantitative RT-PCR of early neural genes in embryos over time (A, B) or in ectodermal explants (C, D). Explants are from uninjected (UI) embryos or those injected with mRNA coding for Noggin (Nog), FGF8a, or Nog + FGF8a. Embryos in (A) were collected at the times indicated at top (hours post fertilization = hpf) and explants were collected at stage 11.5 (C) or 17 (D). *Ef1a *expression is a loading control. Samples without MMLV RTase added were used as RT- controls (far right). (E) Immunohistochemistry for phosphorylated Histone H3 of ectodermal explants dissected from embryos injected with mRNA coding for Nog, FGF8a, or Nog+ FGF8a and cultured to stage 17. Explants injected with Nog+ FGF8a are up to 1.7 fold (cm) bigger than those from uninjected embryos. Graph is showing the number of cells marked with pH3 per mm^2 ^expression in n = 10 explants. Nog+ FGF8a caps have 1.8 fold more pH3 per count area than uninjected explants (p=.0001). UI indicates explants that were dissected from uninjected embryos, WE is whole embryo.

The early neural genes *sox2, sox3, geminin, foxD5a, soxD *and *zicr1 *are expressed broadly in the neuroectoderm at the time of neural induction in response to neural inducing signals [31,35-39]. We compared their expressions at stage 8 (mid-blastula transition, 7+ hpf), through the onset of gastrulation and neural induction (stage 10.5, 10 hpf) and in neurula embryos (stage 17, 24 hpf) (Figure 1A). The maternal genes, *soxD*, *sox3 *and *geminin *are expressed strongly at 7 hpf whereas *foxD5a*, which is also maternally expressed, is expressed at low levels at 7 hpf with levels increasing at 8 hpf and peaking at 10 hpf. The expression levels of the zygotically expressed gene *zicr1 *are fairly constant whereas *sox2 *levels increase from 7 to 24 hpf. Both FGF8 and the BMP antagonist, *noggin *are expressed at 7 hpf preceding neural induction. All of the genes are expressed prior to the onset of gastrulation with zygotic *FGF8 *expressed prior to *foxd5α *and *sox2 *expression indicating that it may play a role in the induction of these two genes but not the others.

To determine if the early neural genes respond to BMP antagonism and/or FGF signaling, we overexpressed Noggin, FGF8a or a combination of both and analyzed the expression of the early neural genes and the proneural gene *neurogenin *(*ngnr1*). We used FGF8a because its misexpression was reported to induce neural tissue in explants without inducing mesoderm [21], and because FGF8a overexpression does not induce *Xbra *expression in whole embryos (Additional File 1, Fig. S1A). Embryos were injected at the 1-cell stage, collected at stage 10 and assayed by RT-PCR. Expression of *soxD*, *sox3*, *zicr1*, *foxd5α *and *ngnr1 *was enhanced by overexpression of Noggin or FGF (Figure 1B). However, expression of *gem and sox2 *was unchanged (Figure 1B) indicating that BMP antagonism and FGF8 are not sufficient to enhance their expression at this stage.

To determine if FGF8a signaling is sufficient to activate or maintain the expression of the early neural genes, we analyzed their expression in gastrula (st. 11.5) and neurula (st. 17) stage ectodermal explants (Figure 1C and 1D) in response to Noggin, FGF8a and Nog+FGF8a. Genes expressed at high maternal levels (*sox3, soxD *and *gem) *were not examined at stage 11.5. As shown in prior studies, BMP inhibition induced the expression of neural progenitor markers (*sox2*, *sox3 *and *geminin*) and the other early neural markers, (*soxD, zicr1, foxd5α*) by st. 17. At stage 11.5, FGF8a induced *zicr1, foxd5α *and *sox2 *expression and by stage 17 induced neuron formation; however, it also induced the expression of the pan-mesodermal marker *xbra *(Figure 1C, D) and the dorsal mesoderm marker and BMP antagonist, *noggin *(Figure 1D). Therefore, it is possible that FGF8a alters neural gene expression indirectly via signals from the dorsal mesoderm.

Explants from embryos co-injected with Noggin and FGF8a are comprised of neural progenitors (*sox2+*, *sox3*+ and *gem+*) and neurons (*n-tub*+) (Figure 1D) and have a distinct morphology; by stage 17, they are ~ 2-fold larger than UI, Noggin or FGF8a caps indicating either increased cell size or proliferation (Figure 1E). To determine if the increase in explant size was due to increased proliferation, we performed immunohistochemistry to detect phosphorylated Histone H3 (Figure 1E), an indicator of mitosis. Explants from embryos injected with Nog and FGF8a mRNA had an average of 1.8 fold more proliferating cells per square millimeter than uninjected explants (Figure 1E, p = .0001, Student's T-Test, n = 10). To verify that the size difference was not due to the presence of large migratory neural crest cells with extended processes, we assayed for the expression of the neural crest marker, *slug*. These explants did not express *slug *(data not shown), but did express the epidermal and mesodermal marker *vent2*, the neuronal marker *n-tub *and the proliferating progenitor markers *sox2, sox3 *and *gem *(Figure 1D). One possible explanation for the mixed cell population in these large explants is that their fate varies with the level of Noggin or FGF received.

### Ectoderm is competent to respond to BMP inhibition and FGF signaling prior to the onset of gastrulation

**Figure 2:**
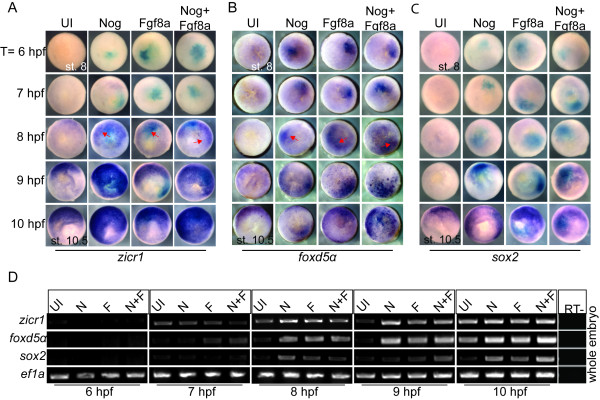
**BMP inhibition and FGF signaling prematurely induce the expression of early neural genes**. (A-C) WISH for *zicr1*, *foxd5α *and *sox2 *of embryos injected with mRNA coding for Nog, FGF8a or Nog + FGF8a and *lacZ *mRNA (cyan) and collected at stage 8 (t = 6 hpf) and each subsequent hour after until stage 10.5 (t = 10 hpf) when cultured at room temperature. Red arrows indicate earliest onset of expression. All images are animal pole view with dorsal to the top. (D) RT-PCR of whole embryos dissected from uninjected embryos (UI) or embryos injected with Nog, FGF8a or Nog+FGF8a. Embryos were collected as in A-C. Genes analysed are indicated on left side, treatment on top, time of collection below panel. *ODC *used for loading control. All images are animal pole view with dorsal to the top.

To determine when ectodermal cells are competent to respond to FGF8a signaling or BMP inhibition by Noggin, we assayed for the expression of the early neural genes, *zicr1*, *foxd5a *and *sox2 *prior to MBT (6 hpf) and until the onset of gastrulation and neural induction (10 hpf). Embryos were injected with mRNA coding for Noggin, FGF8a or Noggin+ FGF8a in 1 of 2-cells and collected at 6 hpf and every hour after until stage 10.5 (10 hpf). We performed whole-mount in situ hybridization (WISH) and semi-quantitative RT-PCR to detect the expression of *zicr1*, *sox2 *and *foxd5α *(Figure 2A-D), which were expressed between 7 and 10 hpf (Figure 1A) in embryos and induced by both Noggin and FGF8a by stage 11.5 in ectodermal explants (Figure 1C). Endogenous expression of *zicr1 *was first detected by WISH at 8 hpf (Figure 2A) and by RT-PCR at 7 hpf. BMP inhibition by Noggin enhanced expression of *zicr1 *at 8 hpf (Figure 2A red arrows, Figure 2D) and by a truncated BMPR (tBR) at 7 hpf (Additional File 2, Figure S2A, C). FGF8a also increased *zicr1 *expression by 8-9 hpf (Figure 2A, D, Additional File 2, Figure S2C). Low levels of *zicr1 *were detected at all stages tested, thus neither BMP inhibition nor FGF signalling induced expression prematurely. In contrast, *foxd5α *expression was induced by Noggin and FGF8a by 8 and 7 hpf (Figure 2B red arrows, 2D), respectively, whereas endogenous dorsal-specific expression was first detected at 9 hpf. *Sox2 *expression was also induced prematurely at 8-9 hpf (Figure 2C, D), and expanded by FGF8a, Noggin and tBR at 10 hpf (stage 10.5) (Figure 2C, Additional File 2, Figure S2B). In summary, *foxd5α *and *zicr1 *are induced and expanded, respectively, in the ectoderm in response to BMP inhibition and FGF8a signalling by 8 hpf, whereas *Sox2 *expression is not significantly altered in response to FGF8a signaling until much later after the onset of endogenous expression (10 hpf). These experiments indicate that early neural genes are induced by Noggin by 8 hpf and respond to FGF8a at different times.

### FGF signaling is required for the induction of *sox2 *and *foxd5a *expression

**Figure 3:**
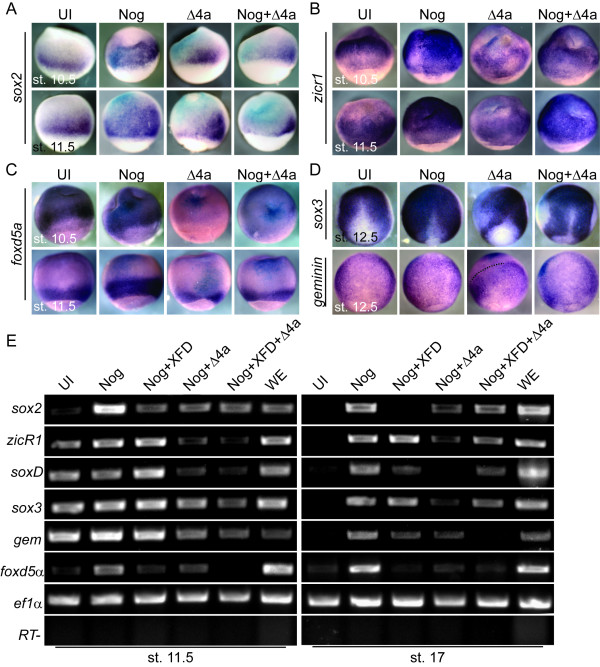
**FGF signaling is required for the induction of *sox2 *and *foxd5α *expression in the neural plate and the maintenance of other early neural genes**. WISH for (A) *sox2*, (B) *zicr1*, (C) *foxd5α*, and (D) *sox3 *and *gem *of embryos that were either uninjected (UI) or injected with Noggin (Nog), dominant negative FGFR4a (Δ4a), or Nog + Δ4a and were collected at stages 10.5 and 11.5 (A-C) or 12.5 (D). All embryos are dorsal view with anterior to the top. The dashed line separates the domain of *gem *expression (right) from that inhibited by Δ4a (top left). (E) RT-PCR of ectodermal explants for genes indicated on the left side of panel. Explants were dissected from embryos that were either uninjected (UI) or injected with mRNA coding for Nog, Nog + dominant negative FGFR1 (XFD), Nog+Δ4a, or Nog+XFD+Δ4a.

In *Xenopus*, the induction of neural tissue occurs at the onset of gastrulation in response to BMP inhibition, and this induction may be dependent on instructive FGF signaling. To determine if BMP inhibition and/or FGF signaling are required for neural tissue formation, we analyzed the expression of the early neural genes in response to constitutively active BMP signaling and the loss of FGF signaling. In support of the neural default model, overexpression of a constitutively active BMP receptor (Alk3) inhibited the expression of the six early neural genes at the onset of neural induction (Additional File 3, Fig. S3) and Noggin expanded their expression (Figure 3A-D and data not shown), suggesting that the expression of all early neural genes requires BMP inhibition. In contrast, each of the early neural genes responded differently to loss of FGF signaling. To interfere with FGF signaling, we used dominant negative FGFR4a (Δ4a) because it is the receptor through which FGF8 stimulates neuron formation [40] and was deemed more effective at blocking neural development than dominant negative FGFR1 (XFD) [25,41]. Surprisingly, although FGF8a induced *soxD *expression in explants, Δ4a overexpression had no effect on *soxD *expression in embryos (data not shown). However, Δ4a expression inhibited *sox2 *(n = 42/62) and *foxd5a *(n = 23/25) expression and reduced *zicr1 *expression (n = 20/30) at stage 10.5, and *sox3 *and *gem *expression (*sox3*, n = 56/63 and *gem *12/12) at stage 12 (Figure 3A-D). Since FGF signaling inhibits BMP signaling via phosphorylation of Smad1 [28], we wanted to determine if the Δ4a phenotypes could be rescued by Noggin, or in other words, were due to increased BMP signaling. *Zicr1, sox3 *and *gem *expression were rescued by overexpression of Noggin (Figure 3B, n = 26/26, 3D, n = 17/21, n = 15/20) and therefore not dependent on FGF signaling. However, *sox2 *and *foxd5a *expression were not rescued by overexpression of Noggin (Figure 3A, C) indicating an independent role for FGF signaling. These data indicate that FGF signaling is required for the expression of *sox2 *and *foxd5α*.

We next tested whether FGF signaling through FGFR4a is required for the expression of early neural genes in explants in which BMP signaling is inhibited. We overexpressed Δ4a in ectodermal explants neuralized by Noggin and performed semi-quantitative RT-PCR. As in embryos, FGF signaling was required for the expression of *sox2 *and *foxd5α *in neuralized mid-gastrula or neurula explants (Figure 3E). Although, *zicr1 *and *soxD *expression were reduced by Δ4a in *noggin*-injected stage 10.5 embryos (Figure 3A, data not shown), their expression was greatly reduced or inhibited by Δ4a expression in *noggin*-injected explants (Figure 3E). In fact, by neurula stage, the expression of all of the early neural genes was greatly reduced or lost in explants co-injected with Noggin and Δ4a. XFD inhibited *sox2 *and *foxd5a *expression and by st. 17 also reduced *soxD *and *gem *expression. In summary, the explant RT-PCR data and WISH embryo data (Figure 3A-D) indicate that *soxD, sox2, zicr1 *and *foxd5α *require FGF signaling for robust expression at the gastrula stage, whereas *sox3 *and *gem *require FGF signaling for the maintenance of their expression in explants after induction by BMP inhibition.

### FGF8a induces neurons in the presence of BMP signaling and epidermis formation

**Figure 4:**
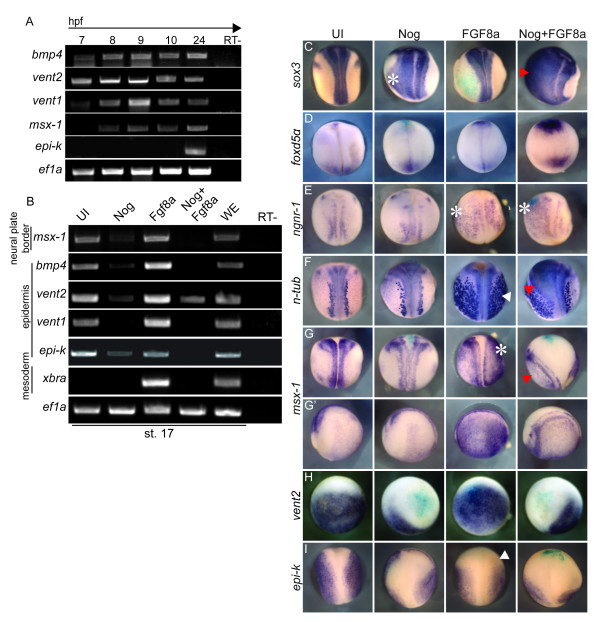
**FGF signaling induces neuron formation despite the presence of BMP signaling**. (A) RT-PCR of embryos collected at the hpf indicated at the top. Primers are indicated on the left side of the panel. *Ef1a *was used as a loading control and samples without MMLV RTase added were used as RT- controls (far right). (B) RT-PCR of stage 17 ectodermal explants dissected from uninjected embryos (UI) or embryos injected with mRNA coding for Nog, FGF8a, or Nog+ FGF8a. WE is whole embryo control. Primers for markers of the neural plate border (*slug*, *msx-1*), epidermis (*bmp4*, *vent2*, *vent1*, *epi-k*) and mesoderm (*xbra*) and are shown on the left side of the panel. (C-I) WISH for (C) *sox3*, (D) *foxd5α*, (E) *ngnr-1*, (F) *n-tub*, (G, G') *msx-1*, (H) *vent2 *and (H) *epi-k *using embryos that were either uninjected (UI) or injected with either Nog, FGF8a or Nog+ FGF8a and *lacZ *mRNA. All stage 17 embryos are a dorsal view with anterior to the top (C-G, I) and G' embryos are a lateral view of embryos in G with anterior to the left. Embryos in H are stage 10.5 animal pole view with dorsal to the top. Asterisk indicates expansion in the deep layer of the ectoderm; white arrow indicates expansion in both the deep and superficial ectoderm and red arrow indicates expansion in the superficial layer only.

FGF8a induces neural gene expression in explants (Figure 1) and expands their expression in embryos (Figure 2), and FGF signaling is required for the expression of the neural progenitor markers *sox2 *and *foxd5α *in the neural plate (Figure 3) [8,17]. To determine if FGF induces early neural gene expression by repressing BMP signaling, we tested the effect of FGF8a on the expression of BMP, its targets, and epidermis formation. First, we determined the temporal expression of BMP target genes prior to and at the onset of neural induction in embryos (Figure 4A). Embryos were collected as for Figure 1. *Bmp4 *expression was detectable by RT-PCR at 7 hpf, and was increased after the onset of zygotic transcription at 8 hpf (Figure 4A). The direct target of BMP4, *vent2 *[42], was also expressed robustly at 7 hpf, and the expression of *vent1*, a direct Vent2 target [43], followed at 8 hpf. *Msx-1*, a BMP target that is eventually restricted to the neural plate border, was first detected at 8 hpf and the definitive epidermal marker *epi-k *was not expressed in any stages prior to stage 10.5 but is expressed at stage 17 (24 hpf, Figure 4A).

To determine the effect of FGF signaling on BMP signaling and epidermal development, we injected mRNA coding for Noggin, FGF8a, or Nog and FGF8a together, dissected ectodermal explants, cultured them until stage 17 and performed RT-PCR for markers of BMP signaling and epidermal development. In Noggin-expressing explants, the expression of BMP4 and its targets were decreased significantly (Figure 4B). In contrast, FGF8a enhanced the expression of *bmp4*, BMP target gene expression and epidermal formation (Figure 4B) even though it also induced neurons and mesoderm in explants (Figure 1D). In embryos, overexpression of FGF8a inhibited *vent2 *expression at 8 hpf, but expression recovered one hour later (Additional File 1, Fig. S1B). When FGF8a and Noggin were injected together, the expression of all markers except *vent2 *was lost (Figure 4B).

These data indicate that FGF signaling is required for the induction of both *sox2 *and *foxd5α *and for the continued expression of *sox3 *and *gem *(Figure 3). Additionally, overexpression of FGF8a in explants leads to the expression of markers of neural progenitors, neurons and mesoderm and has no overt effect on epidermal development in embryos. To determine the fate of FGF8a- and Nog/FGF8a -injected cells in embryos, we injected mRNA coding for Noggin, FGF8a, or Noggin+FGF8a into 1 of 2 cell embryos, cultured the embryos until neurula stage, and performed WISH for *sox3 *(neural progenitors), *foxd5α *(early neural), *ngnr-1 *(proneural), *n-tub *(neurons), *msx-1*(border cells), *vent2 *(non-neural ectoderm), and *epi-k *(epidermis). Overexpression of Noggin or FGF8a expanded the neural progenitor marker, *sox3 *(Figure 4C, Nog, n = 20/22; FGF8a, n = 19/21) and together, dramatically expanded its expression throughout the entire injected side (n = 20/20). Alone, neither Noggin nor FGF8a affected the expression of *foxd5α *(Nog, n = 13/13; FGF8a, n = 6/9), but together increased *foxd5α *expression (Figure 4D, n = 11/13). Noggin had no effect on proneural (*ngnr-1*, Figure 4E, n = 14/16) or neuronal (*n-tub*, Figure 4F, n = 21/21) gene expression whereas FGF8a (*ngnr-1*, n = 14/14; *n-tub*, n = 20/20) and Nog+ FGF8a (*ngnr-1*, n = 14/14; *n-tub*, n = 20/20) induced ectopic expression of *ngnr-1 *and *n-tub *in lateral and ventral ectoderm (Figure 4E, F). These data confirm previous observations that BMP inhibition is sufficient to induce neural progenitors, but only weakly induces the formation of neurons [44]. Furthermore, FGF8 only weakly expands the neural progenitor population but greatly expands the population of neurons [40]. These data indicate that after induction of neural tissue by BMP antagonists, an additional signal is required to cue progenitors to differentiate into neurons. FGF8a effectively serves as this signal.

Experiments in explants show that FGF8a does not induce the expression of neural genes by inhibiting BMP signaling (Figure 4B). To determine if this is the case in embryos, we injected FGF8a with and without Noggin and assayed for the expression of non-neural ectodermal markers. Noggin repressed the expression of *vent2 *(Figure 4H, Additional File 1, Fig. S1B, n = 21/21) and *epi-k *(Figure 4F, n = 12/12) and reduced but dispersed *msx*-*1 *expression (Figure 4G, G', n = 12/12) in neurula stage embryos. In contrast, even though FGF8a induced ectopic neurons in embryos, it did not repress the expression of non-neural ectodermal or differentiated epidermal markers (Figure 4G-I, *vent2*, n = 27/29; *msx-1*, n = 14/14; *epi-k*, n = 14/22). In fact, ectopic *n-tub *(Figure 4F) expression overlapped with that of *epi-k *and ectopic *msx-1 *in the superficial ectodermal layer (Figure 4 G, I and Additional File 4, Fig. S4). With the addition of Noggin, the ectopic neurons were excluded from the deep layer and did not overlap with epidermis, which is repressed by Noggin (Additional File 4, Fig. S4).

In summary, FGF8a expands proneural, neuronal, and non-neural domains such that *msx1*, *n-tub *and *epi-k *are expressed in the same cells but segregated from proliferating progenitors. Noggin represses epidermal development to induce neural markers whereas FGF8a does not, suggesting that BMP antagonism and FGF signaling do not use the same mechanisms to induce the expression of neural genes.

## Discussion

The long-standing debate over the signals required for neural induction led us to investigate the requirements for the induction and maintenance of expression of the first five genes expressed in the neural plate, which we designate as early neural genes. We have determined that BMP inhibition is necessary but not sufficient for the onset of expression of all of the early neural genes. BMP inhibition is both necessary and sufficient to induce the expression of *zicr1*, but expression of *sox2 *and *foxd5α *in the neural plate also requires signaling through FGFR4a. Additionally, FGF8a signaling is sufficient to prematurely induce or expand expression of *zicr1*, *foxd5α *and *sox2 *in unspecified ectoderm, and to expand the neural tube and induce ectopic neurons at later stages. However, FGF8a has no effect on epidermal development indicating that it does not exert its effect via BMP inhibition. Our results add to the prior known roles of FGF signaling and BMP inhibition in neural gene expression by showing that they are involved in the expression of different early neural genes, and that they act independently of each other.

### BMP signaling is sufficient to inhibit neural gene expression at the onset of neural induction

Many studies have shown that inhibition of BMP signaling by antagonists such as Chordin [45], Noggin [46] and Follistatin [47] induces neural tissue. Additionally, studies have shown that overexpression of known BMP targets such as Msx-1 [48,49], Xvent1 [50] and Xvent2 [51] ventralize *Xenopus *embryos. Although BMP signaling is known to repress neural development and induce epidermal genes [52], studies have yet to show if BMP signaling inhibits the onset of expression of neural specification genes (early neural genes) in dorsal ectoderm or if the induction of their expression is dependent on other signals. We overexpressed a constitutively active BMP receptor (CaBMPR) and showed that active BMP signaling repressed the onset of zygotic expression of *sox2*, *zicr1*, *sox3*, *gem*, *and foxd5α*. CaBMPR also repressed the expression of the neural inducer, *soxD*, by the end of gastrulation (Additional File 3, Fig. S3). Since *sox3 *and *gem *are expressed pan-ectodermally until stage 11.5, and thus initially overlap with *bmp4 *and *vent2 *expression, it is surprising that CaBMPR repressed their expression. Why aren't they repressed by BMP signaling in ventral cells in early gastrulae? One possibility is that repression requires both Vent1 and Vent2, which are not expressed together until mid-gastrula stages unless prematurely activated by overexpression of CaBMPR.

### Overexpression of FGF and BMP antagonists induce *zicr1 *and *foxd5a *expression prematurely

Prior experiments led to the conclusion that BMP antagonism is not sufficient for the induction of neural tissue in chick epiblast or ventral ectoderm of frogs [16,18]. We argue that BMP inhibition is sufficient for the induction of some early neural genes in unspecified ectoderm. Through our analysis of early neural gene expression, we find that the initiation of *sox2 *and *foxd5α *expression requires FGF signaling in addition to inhibition of BMP signalling. This supports the conclusions of previous studies that demonstrated that an FGF4 morpholino inhibited the expression of *sox2 *in ectoderm in response to the dominant negative R-Smad, Smad5-sbn [17], but contradicts recent studies that demonstrated that FGF is only required for expression of *sox2 *in the circumblastoporal region [8]. However, we also show that Noggin is sufficient to induce the expression of *foxd5α *prematurely in unspecified ectoderm indicating that FGF is present in the ectoderm at this time (Figure 2A, B). This is in contrast to studies in: (1) chick embryos in which inhibition of BMP signaling is not sufficient to induce the expression of *sox2 *in non-neural tissue even in the presence of FGF8 [18] and; (2) frog embryos in which BMP inhibition by overexpression of Smad6 was insufficient to induce *sox2 *in ventral ectoderm unless TGF-β signaling was also inhibited [15,16] or eFGF added [15]. We argue that differences in developmental timing, tissue competence and experimental approaches may account for the different conclusions drawn. Specifically, our experiments differ from past studies in that we assay for the expression of neural genes prior to and contemporaneous with neural ectoderm specification whereas previous studies tested the ability of BMP inhibition to convert the fate of epiblast or epidermal cells permanently by testing gene expression in later stage embryos. In past studies, it is possible that BMP inhibition allowed for transient expression of neural genes at the onset of neural induction, but this expression was not maintained and therefore not detected. Our studies considered with these studies indicate that inhibition of BMP induces neural genes in unspecified ectoderm of early gastrula embryos but this induction is transient and a second signal such as a TGFβ inhibition [16] or FGF signaling [15] found only in dorsal ectoderm is required to maintain their expression.

### FGF signaling is required for the induction of *sox2 *and *foxd5a *expression in the neural plate

FGF8a signaling is sufficient to expand *sox3 *expression and induce ectopic neurons in embryos (Figure 1 and Additional File 4, Figure S4). Furthermore, FGF signaling is required for the expression of *sox2 *and *foxd5α*. These two genes are reported to be essential for the differentiation of neural ectoderm [53,54] indicating that FGF signaling is also essential for neural development. The loss of expression of these genes is not rescued by antagonism of BMP signaling by Noggin indicating that FGF signaling is instructive or permissive and does not induce *sox2 *or *foxd5a *expression by inhibiting BMP signaling. However, the requirements for FGF are different for other early neural genes. For example, although misexpression of dominant negative FGFR4a reduces *zicr1 *expression at st. 10.5, this loss is rescued by Noggin. Therefore, FGF signaling induces *zicr1 *expression by either inhibiting BMP signaling [28], or sensitizing the ectoderm to BMP inhibition. The latter hypothesis is supported by our *in vivo *experiments (Additional File 4, Figure S4), which show that subsequent to FGF8a overexpression, the ectoderm is competent to express both neural and epidermal markers in the same tissue. Contrary to this theory, recent studies in mouse embryonic stem cells suggest that unlike mFGF2, which promotes stem-like renewal of multipotent epiblast cells, mFGF8 creates a population of specified transient neural progenitors [55]. However, in these studies the neural progenitors were unable to differentiate into neurons as do FGF8^+ ^*Xenopus *ectodermal cells (Additional File 4, Figure S4) suggesting that xFGF8a has additional functions in development [55].

*Sox3 *and *gem *are unaffected by knock down of FGF signaling via Δ4a at early gastrula stages, but their expression is lost by the end of gastrulation indicating that although these genes do not require FGF signaling for induction, FGF is necessary for maintenance of their expression (Figure 3D). This is supported by the loss of their expression in stage 17 but not stage 11.5 explants in response to Δ4a. Furthermore, Sox3 directly activates the expression of *gem *[56], and Sox3 and Geminin may both activate expression of themselves forgoing the necessity for an instructive signal such as FGF.

Finally, *soxD *expression has different regulatory requirements than all of the other early neural genes. Although it is induced by FGF8a overexpression in explants (Figure 1D), knock down of FGF signaling via Δ4a has no effect on *soxD *expression in embryos (data not shown) and past studies showed that knock down of FGF13 signaling also had no effect [57]. Furthermore, *soxD *is induced by BMP inhibition (Figure 1D) [37], but constitutive BMP signaling has no effect on the induction of *soxD *at stage 10.5, only on its maintenance at stage 12 (Additional File 3, Fig. S3).

### FGF8a induces and expands neural genes in the presence of mesoderm without inhibiting BMP signaling and epidermal development

BMP inhibition by misexpression of Noggin predominantly induces neural progenitors at the expense of epidermis (Figure 1D, 4B). In contrast, FGF8a overexpression induces the formation of neurons (Figure 1A) without inhibiting epidermal formation (Figure 4 and Additional File 4, Fig. S4), suggesting that FGF8a acts in a pathway independent of BMP inhibition to induce neural gene expression and neuron formation. However, induction of mesoderm by FGF8a is dependent on BMP signaling as it is blocked with the co-injection of *noggin *mRNA.

FGF8a signaling induces the premature expression of neural genes in ectoderm and ectodermal explants in cells that are also expressing epidermal genes (Figure 4 and Additional File 4, Figure S4). Although previous studies showed that the superficial layer of the ectoderm is less competent to respond to neuronal inducing signals [58], we found that FGF8a induces *n-tub *positive neurons in both the superficial and deep ectodermal layers that overlap with *epi-k *expressing cells (Additional File 4, Figure S4). One interpretation is that FGF8a maintains the competency of the superficial layer to undergo neurogenesis. It is also possible that FGF8a induces neurons without increasing the progenitor pool, but this is hard to envision without a loss of epidermal gene expression. Another possibility is that FGF8a overexpression induces mosaic cell fates in explants based on the levels of protein bound by each cell. Our data support the hypothesis that overexpression of FGF8a maintains cellular competence since the FGF8 injected cells form many tissue types indicated by markers of epidermis, neurons and neural progenitors.

## Conclusions

In toto, this research shows that FGF signaling induces two early neural genes, *sox2 *and *foxd5α*, independent of epidermal development. These data refute prior studies because they show that both BMP inhibition and FGF signaling are sufficient to induce neural tissue as marked by early neural genes in *Xenopus *ectoderm. Finally, we have shown that the regulation of early neural genes is unique even within gene families (e.g. *soxB1*) and therefore, conclusions about the requirement for FGF signaling in neural induction may be gene specific. Although previous models for neural induction stated that either BMP inhibition was sufficient or FGF signaling was required for neural induction, here we show that in *Xenopus*, there is no neural induction regulatory module that can explain the induction of all early neural genes; *sox2 *and *foxd5α *require FGF signaling for expression but *sox3*, *geminin *and *zicr1 *do not.

## Methods

### Embryo culturing and manipulations

*Xenopus laevis *embryos were obtained using standard methods [59] and staged according to Nieuwkoop and Faber (1994). Animal ectodermal explants were isolated from stage 8-9 embryos, cultured in 0.75 × Normal Amphibian Medium (NAM), and were collected between stages 11.5 and 17 based on sibling embryos.

### mRNA Synthesis and Microinjection

Synthetic capped mRNA was made by *in vitro *transcription using mMessage mMachine kits (Ambion). For explant and gain of function assays, 25 pg *noggin *mRNA [60,61], 0.5ng of tBR [62] mRNA and 0.3 ng of *lacZ *or 0.3 ng *GFP *mRNA was injected into the animal pole of a 1 or 2-cell embryo with or without 0.5 ng of dominant negative *Xfgfr1 *mRNA (*XFD*) [63], 0.5-1.5 ng of dominant negative *XFGFR4a *mRNA (*Δ4a*) [25], or 0.2 ng of *FGF8a *mRNA. Embryos were cultured until stages 8-17 and analyzed by WISH or reverse transcription- polymerase chain reaction (RT-PCR).

### RT-PCR

Semi quantitative RT-PCR was performed as described [64] with some modifications. Prior to reverse transcription, 1 μl of isolated RNA was used for PCR with primers for *ef1α *(XMMR) to determine if there was DNA contamination. To make cDNA, 10 μl of isolated RNA was mixed with 1 μl random hexamers and heated to 65° for five minutes then incubated at 42° with MMLV for 1 hour. RT minus samples underwent the same treatment minus MMLV reverse transcriptase. Primers used: *sox2*, *sox3*, *geminin*, *zicr1*, *soxD, foxd5α*, *vent2*, *vent1*, *msx1*, *bmp4*, *epi-k*, *nog*, *fgf8*, *xbra*. RNA was extracted from a minimum of two whole embryos or 10 explants per stage/treatment.

### WISH and β-galactosidase assay

Whole mount in situ hybridization (WISH) was performed as described [65,66] and with the following modifications: embryos were not treated with proteinase K, triethanolamine, or acetic anhydride, and pre-hybridization was shortened to one hour. After an overnight hybridization, embryos were washed in 1× maleic acid buffer (MAB) and then incubated in digoxigenin antibody at room temperature for four hours followed by three times 15 minute washed with 1× MAB, and an overnight incubation at 4° in 1× MAB. Finally, embryos were either fixed in 4% formaldehyde with 0.5% acetic acid, and 2× SSC, or Bouin's fixative. For lineage tracing, *β*-galactosidase activity was visualized with X-gal (Research Organics). We generated digoxigenin labeled mRNA probes for *sox2 *[36], *sox3 *[67], *vent1 *[50], *vent2 *[51,68-70], *geminin *[39], or *GFP *[71], *zicr1 *[36], *soxD *[37], *foxd5α *[35], *msx-1 *[72], *n-tub *[73], and *epi-keratin *[74].

## Authors' contributions

CDR performed the experiments, participated in the study design and drafted the manuscript with ESC. GSF performed experiments and embryo phenotype analysis and photography. ESC conceived of the study, participated in its design and coordination and helped to draft the manuscript. All authors read and approved the final manuscript.

## Supplementary Material

Additional file 1 **Fig. S1. FGF8a does not induce *Xbra *expression and transiently inhibits *vent2 *expression**. (A) WISH for *xbra *using embryos that were uninjected (UI) or injected with mRNA coding for FGF8a or Nog+ FGF8a with *lacZ *(cyan) as a tracer and collected at stage 8 and every hour after until stage 10. Embryos were cultured at room temperature. There is no ectopic induction of *Xbra *expression in the injected cells at any stage. (B) WISH for *vent2 *using embryos that were uninjected (UI) or injected with mRNA coding for Nog, FGF8a or Nog+ FGF8a with *lacZ *(cyan) as a tracer and collected at stage 8 (6 hpf) and every hour after until stage 10.5 (10 hpf). Embryos were cultured at room temperature. At 8 hpf *vent2 *expression is decreased in FGF8a injected embryos but expression rebounded by 9 hpf. Click here for file

Additional file 2 **Fig. S2. BMP inhibition and FGF signaling induce the expression of early neural genes**. (A-B) WISH for *zicr1 *and *sox2 *of embryos injected with mRNA coding for tBR, FGF8a or tBR + FGF8a and *lacZ *mRNA (cyan) and collected at stage 8 (t = 6 hpf) and each subsequent hour after until stage 10.5 (t = 10 hpf) when cultured at room temperature. (C) RT-PCR of ectodermal explants dissected from uninjected embryos (UI) or embryos injected with tBR, FGF8a or tBR+FGF8a. Genes analysed are indicated on left side, treatment on top, time of collection below panel. *ODC *used for loading control. All images are animal pole view with dorsal to the top. Click here for file

Additional file 3 **Fig. S3. CaBMPR is sufficient to repress the expression of early neural genes in gastrulae**. (A-F) WISH of embryos stages 10.5 (A-E) or 12 (F) for genes as indicated next to each panel. Embryos were either uninjected (UI) or injected with constitutively active BMP receptor Alk3 (*CaBMPR*) and *lacZ *mRNA. Arrowhead points to site of injection. Pictures shown are representative of majority phenotype: *sox2 *(st. 10.5, n = 35/56; st. 12.5, n = 22/30), *zicr1 *(st. 10.5, n = 27/27; st. 12.5, n = 26/26), *soxD *(st. 10.5, n = 18/19; st. 12.5, n = 27/27), *sox3 *(st. 10.5, n = 62/70; st. 12.5, n = 39/58), *geminin *(st. 10.5, n = 14/16; st. 12.5, n = 22/26), and *, foxd5α *(st. 10.5, n = 25/30; st. 12.5, n = 14/20). Click here for file

Additional file 4 **Fig. S4. FGF8a induces *n-tub *positive neurons in the same tissue layer as *epi-k *positive epidermis**. Bisections of stage 17 whole embryos show that Nog expands *sox3 *expression in the deep layer (marked by asterisk) and represses epidermal gene expression *n-tub *expression. FGF8a overexpression can expand *n-tub *expression in both the deep and superficial layer where epidermal genes (*epi-k*) are expressed (white arrow). Nog+ FGF8a only expands *sox3 *and *n-tub *in the superficial layer. Click here for file
