# Supplementary figures and images for: The response of early neural genes to FGF signaling or inhibition of BMP indicate the absence of a conserved neural induction module
Source: BMC Dev Biol. 2011 Dec 15;11:74. doi: 10.1186/1471-213X-11-74 (PMC3271986; doi:10.1186/1471-213X-11-74)

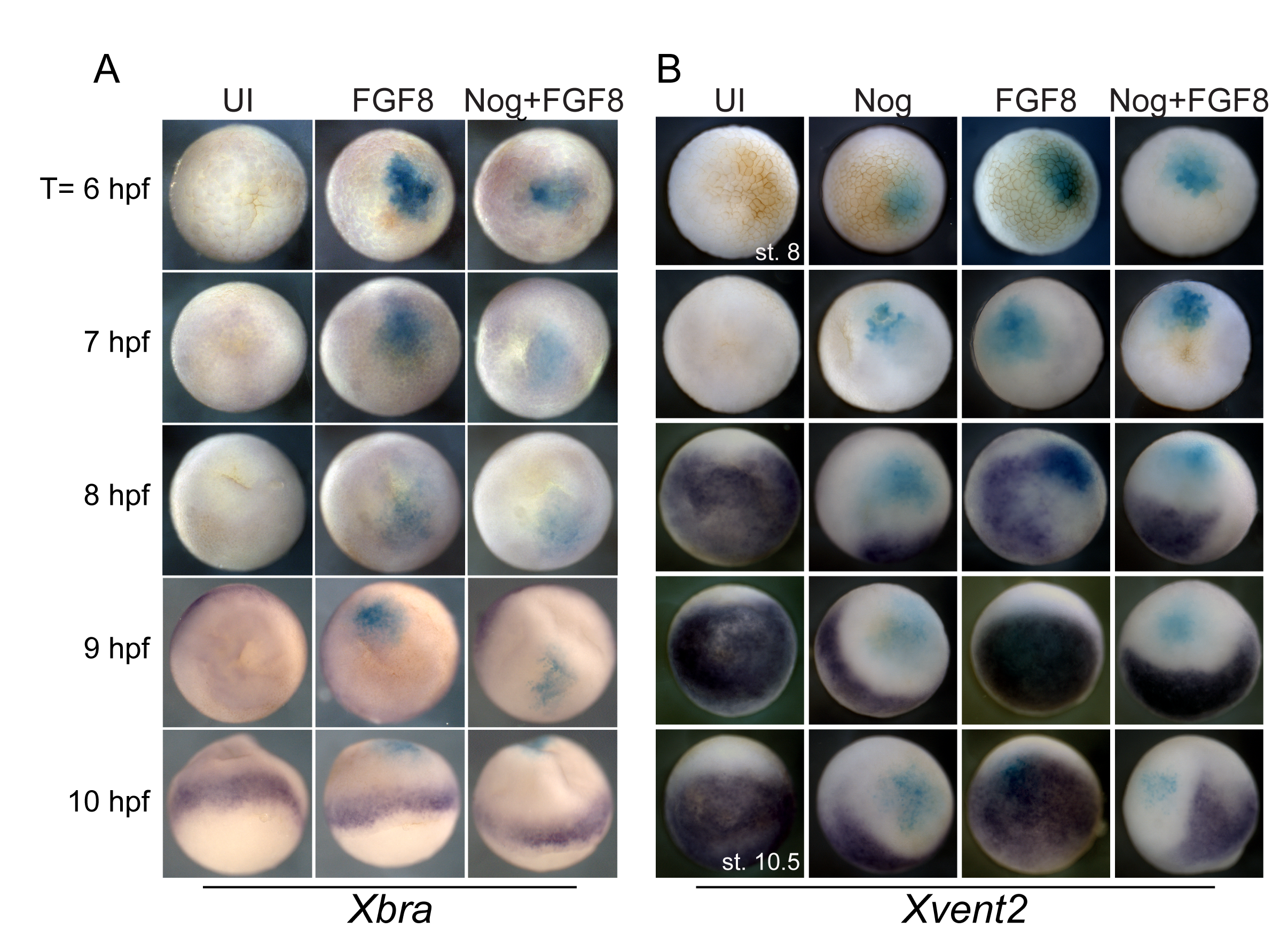

Supplement: Additional file 1 — Fig. S1. FGF8a does not induce Xbra expression and transiently inhibits vent2 expression. (A) WISH for xbra using embryos that were uninjected (UI) or injected with mRNA coding for FGF8a or Nog+ FGF8a with lacZ (cyan) as a tracer and collected at stage 8 and every hour after until stage 10. Embryos were cultured at room temperature. There is no ectopic induction of Xbra expression in the injected cells at any stage. (B) WISH for vent2 using embryos that were uninjected (UI) or injected with mRNA coding for Nog, FGF8a or Nog+ FGF8a with lacZ (cyan) as a tracer and collected at stage 8 (6 hpf) and every hour after until stage 10.5 (10 hpf). Embryos were cultured at room temperature. At 8 hpf vent2 expression is decreased in FGF8a injected embryos but expression rebounded by 9 hpf. [file 1471-213X-11-74-S1.TIFF]

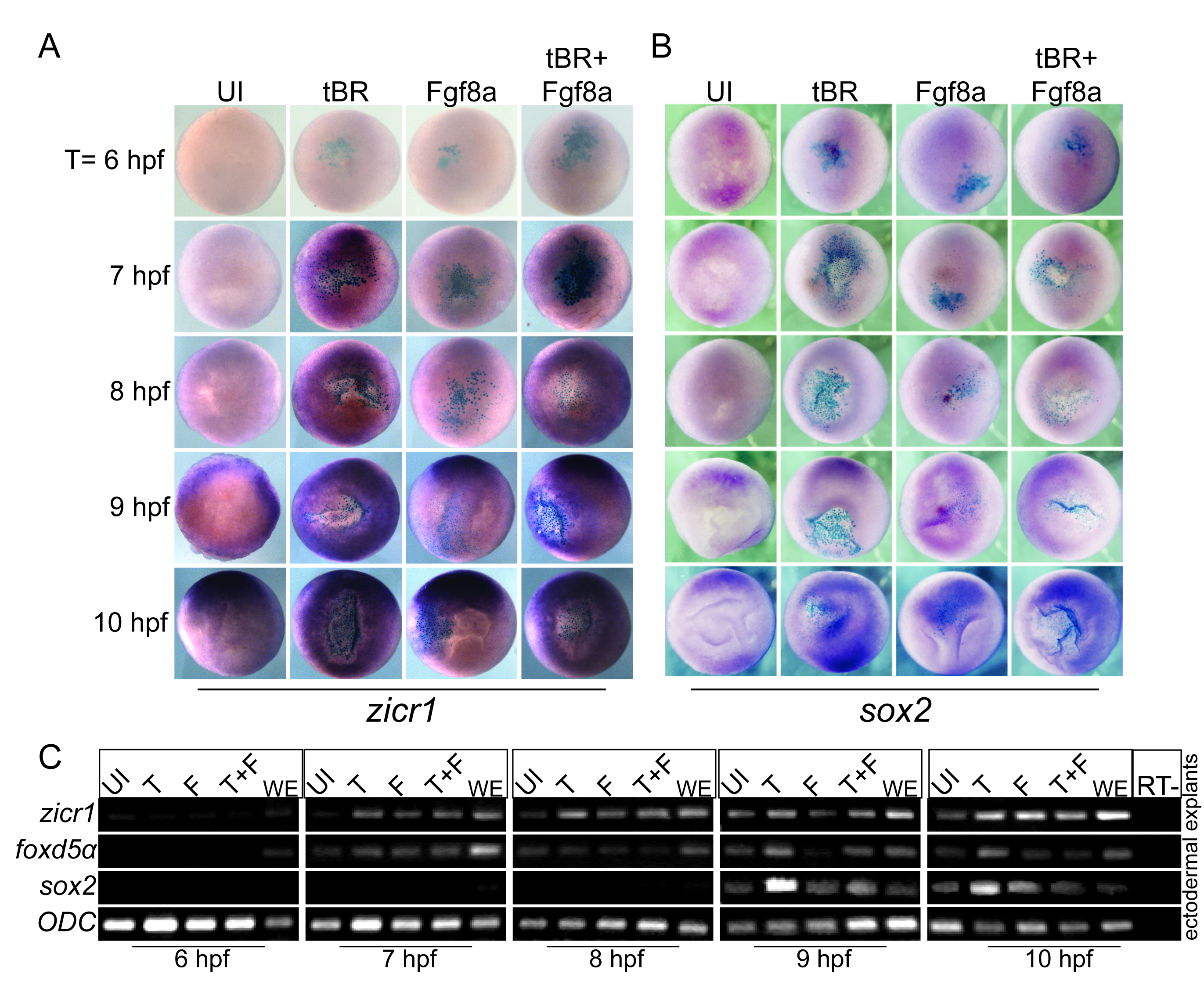

Supplement: Additional file 2 — Fig. S2. BMP inhibition and FGF signaling induce the expression of early neural genes. (A-B) WISH for zicr1 and sox2 of embryos injected with mRNA coding for tBR, FGF8a or tBR + FGF8a and lacZ mRNA (cyan) and collected at stage 8 (t = 6 hpf) and each subsequent hour after until stage 10.5 (t = 10 hpf) when cultured at room temperature. (C) RT-PCR of ectodermal explants dissected from uninjected embryos (UI) or embryos injected with tBR, FGF8a or tBR+FGF8a. Genes analysed are indicated on left side, treatment on top, time of collection below panel. ODC used for loading control. All images are animal pole view with dorsal to the top. [file 1471-213X-11-74-S2.TIFF]

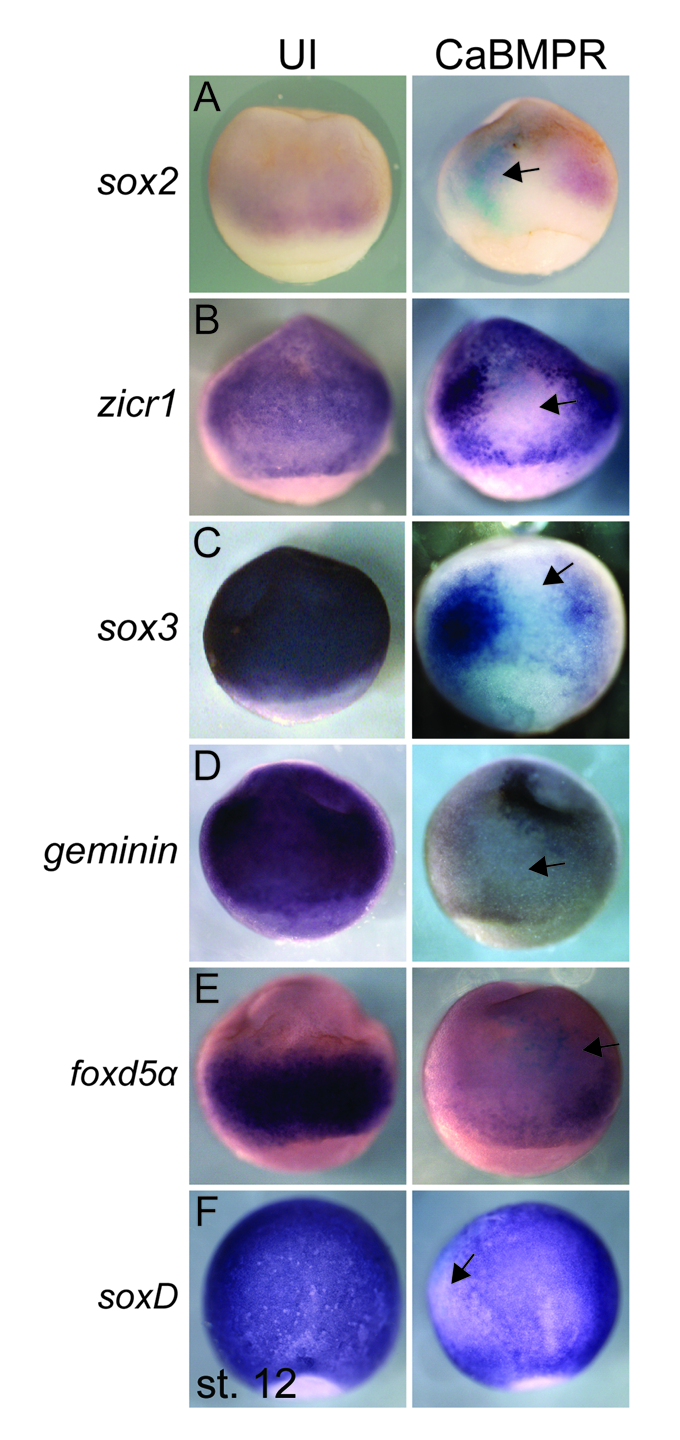

Supplement: Additional file 3 — Fig. S3. CaBMPR is sufficient to repress the expression of early neural genes in gastrulae. (A-F) WISH of embryos stages 10.5 (A-E) or 12 (F) for genes as indicated next to each panel. Embryos were either uninjected (UI) or injected with constitutively active BMP receptor Alk3 (CaBMPR) and lacZ mRNA. Arrowhead points to site of injection. Pictures shown are representative of majority phenotype: sox2 (st. 10.5, n = 35/56; st. 12.5, n = 22/30), zicr1 (st. 10.5, n = 27/27; st. 12.5, n = 26/26), soxD (st. 10.5, n = 18/19; st. 12.5, n = 27/27), sox3 (st. 10.5, n = 62/70; st. 12.5, n = 39/58), geminin (st. 10.5, n = 14/16; st. 12.5, n = 22/26), and , foxd5α (st. 10.5, n = 25/30; st. 12.5, n = 14/20). [file 1471-213X-11-74-S3.TIFF]

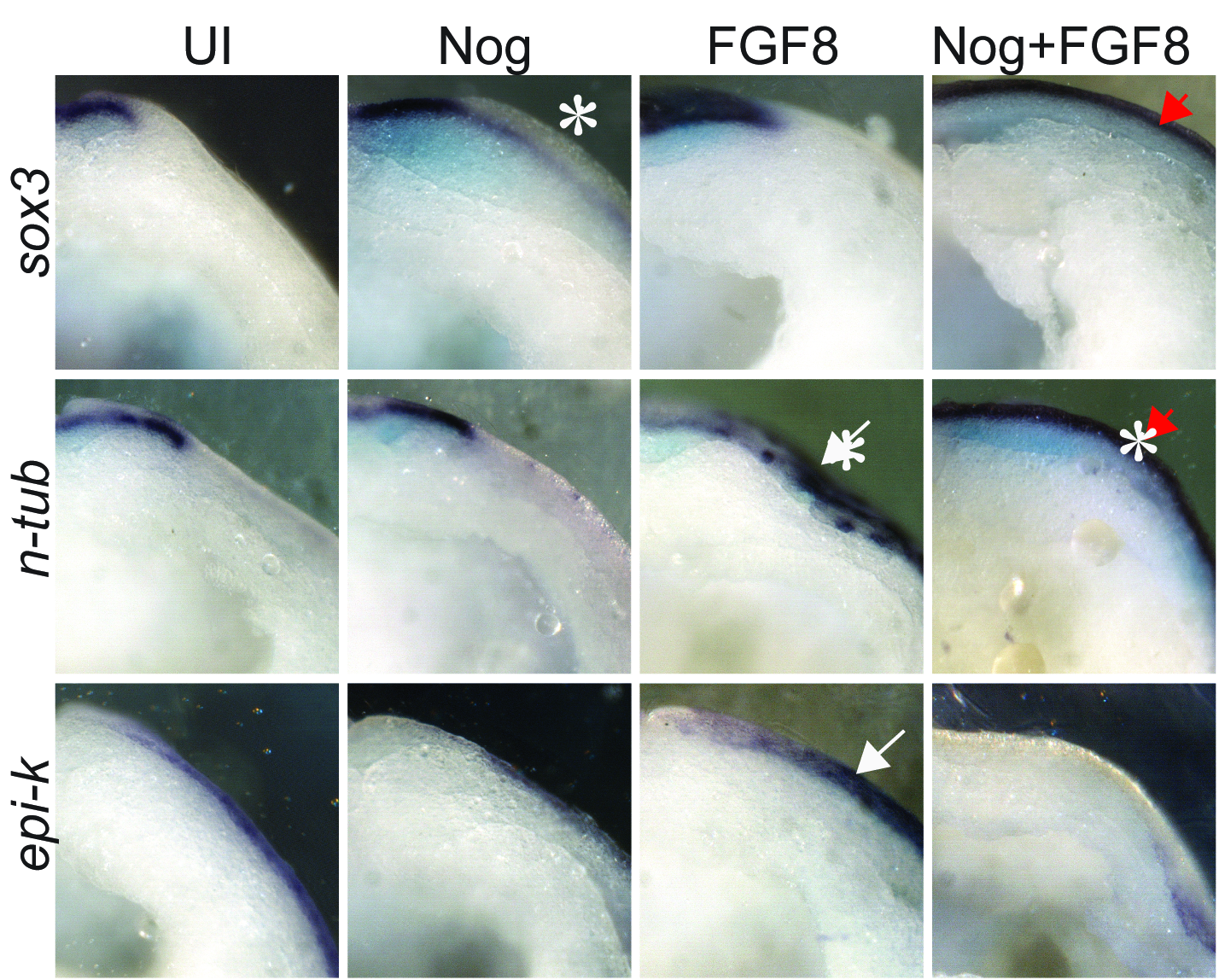

Supplement: Additional file 4 — Fig. S4. FGF8a induces n-tub positive neurons in the same tissue layer as epi-k positive epidermis. Bisections of stage 17 whole embryos show that Nog expands sox3 expression in the deep layer (marked by asterisk) and represses epidermal gene expression n-tub expression. FGF8a overexpression can expand n-tub expression in both the deep and superficial layer where epidermal genes (epi-k) are expressed (white arrow). Nog+ FGF8a only expands sox3 and n-tub in the superficial layer. [file 1471-213X-11-74-S4.TIFF]
